# Droplet digital polymerase chain reaction for the assessment of disease burden in hairy cell leukemia

**DOI:** 10.1002/hon.2932

**Published:** 2021-10-15

**Authors:** Alessandro Broccoli, Carolina Terragna, Laura Nanni, Marina Martello, Silvia Armuzzi, Claudio Agostinelli, Alice Morigi, Beatrice Casadei, Cinzia Pellegrini, Vittorio Stefoni, Elena Sabattini, Lisa Argnani, Pier Luigi Zinzani

**Affiliations:** ^1^ IRCCS Azienda Ospedaliero‐Universitaria di Bologna Istituto di Ematologia “Seràgnoli” Bologna Italy; ^2^ Dipartimento di Medicina Specialistica Diagnostica e Sperimentale Università di Bologna Bologna Italy; ^3^ Hematopathology Unit IRCCS Azienda Ospedaliero‐Universitaria di Bologna Bologna Italy

**Keywords:** BRAF‐V600E mutation, complete response, droplet digital PCR, hairy cell leukemia

## Abstract

*BRAF* ^V600E^ mutation is the pathogenic driver of hairy cell leukemia (HCL) found in the vast majority of cases both at onset and during recurrences. The identification of the mutated allele in blood and marrow correlates with the presence of neoplastic cells and can be considered a marker of active disease. Likewise, the absence of the mutation after treatment may indicate a state of deep response. The *BRAF* ^V600E^ burden was measured by droplet digital polymerase chain reaction (ddPCR) and expressed as fractional abundance in 35 HCL patients at different stages of disease (onset, relapse, complete response [CR] after treatment, long‐term remission) in peripheral blood and/or bone marrow (when available). Mean values of fractional abundance for patients at diagnosis, relapse and response, respectively, were 12.26%, 16.52% and 0.02% in peripheral blood and 23.51%, 13.96% and 0.26% in bone marrow. Four patients out of 6 evaluated at response were molecularly negative for *BRAF* ^V600E^ in peripheral blood. Mean fractional abundance in peripheral blood tested in 14 patients with long lasting CR was 0.05%, and 10 patients were *BRAF* ^V600E^ negative. These preliminary results suggest that ddPCR permits to assess the active tumor burden in HCL at different disease phases and support the hypothesis that some patients in CR qualify for a molecular CR.

## 1 INTRODUCTION

The *BRAF* ^V600E^ mutation is considered the most relevant pathogenic driver of hairy cell leukemia (HCL), being responsible for the neoplastic transformation, the maintenance of the disease and its peculiar phenotype.^1^, ^2^ It can be found in the vast majority of cases (>95%) and at any phase of the disease, either at onset and any subsequent relapse. This is a consequence of the rather simple genetic background of HCL, which is characterized by the acquisition of just a few known mutations during the natural history of the disease, although with the invariable persistence of *BRAF* ^V600E^.^3^, ^4^ This mutation is absent in other lymphoproliferative diseases that may mimic HCL, such as splenic lymphoma with villous lymphocytes and splenomegaly or splenic red pulp lymphoma, thus it is characteristically a hallmark of this disease.^1^, ^5^ Finally, the aberrant BRAF protein encoded by the mutated allele, has proven to be a suitable target for treatment by means of orally available inhibitors (vemurafenib above all), to be applied alone or in combination with other targeted agents like rituximab.^6^, ^7^, ^8^ BRAF blockade is capable of a rapid recovery of peripheral blood cytopenias as well as of the reversal of the hairy cell phenotype of the leukemic cell.^6^

The identification of the mutated allele in peripheral blood (PB) and bone marrow (BM) by means of polymerase chain reaction (PCR) or immunohistochemistry on decalcified tissue obtained trephine biopsy is of importance for diagnosis, although not indispensable.^9^, ^10^, ^11^, ^12^ Likewise, a positive immunohistochemical reaction on marrow tissue is suggestive of disease persistence after an active treatment,^13^ although currently applied response criteria only rely on the resolution of peripheral blood cytopenias and of marrow lymphoid infiltrate in hematoxylin‐eosin staining to assess the depth of the achieved response.^14^, ^15^

Internationally acknowledged guidelines do not currently recommend an assessment of minimal residual disease (MRD) in patients otherwise in complete response (CR), as its impact on patients' outcomes and medium‐to‐long‐term prognosis has never been determined.^12^ Nevertheless, MRD study is now an active field of research in HCL, and the application of immunohistochemistry thresholds for PAX5, CD20, DBA.44, VE1 or ANXA1, as well as flow cytometry assays including the four HCL markers CD11c, CD25, CD103 and CD123 antibodies have been proposed, but never formally validated.^12^

The molecular assessment of the *BRAF* ^V600E^ burden can be an alternative option to detect MRD in residual disease, as well to confirm diagnosis at onset or relapse. Droplet digital PCR (ddPRC) has proven its accuracy in several hematologic disease, including chronic myeloid leukemia and HCL as well, and may be proposed as a tool to detect the presence of the mutation in an investigational setting.^16^

We have implemented a molecular assay based on the use of ddPCR on PB and/or BM in patients with HCL at different phases of their disease, which may individuate a minimal disease burden in patients achieving a CR and that allows monitoring of all CR patients in the post‐treatment phase. Here we present the preliminary results of this exploratory analysis on patients recently treated at our Institution because of a new diagnosis of HCL or relapse, as well as followed‐up after having obtained a CR several years before.^17^, ^18^

## 2 METHODS

### 2.1 Study objective

Given the importance of the *BRAF* ^V600E^ mutation in the pathogenesis of the disease, being present in the vast majority of HCL cases both at onset and at relapse, we have hypothesized that the detection of the mutated allele in either PB or BM could correlate with the presence of neoplastic cells, indicating an active disease, regardless the severity of symptoms or the depth or PB cytopenias. On the other hand, we also postulated that the absence of the mutated allele after treatment (or even in patients with continuous CR) could indicate a state of profound response.

### 2.2 Study overall conduct

In order to demonstrate our hypotheses, we have evaluated the burden of the *BRAF* ^V600E^ mutation in patients at any disease phase: at disease onset in PB and BM (when aspirate was possible), at the time of CR, and in patients with long‐lasting CRs (maintained for at least 5 years) after one course of treatment with cladribine. We have established a laboratory assay based on ddPCR which may individuate a minimal disease burden in patients achieving a CR and that could allow the monitoring of CR patients in the post‐treatment phase. The procedures followed were in accordance with the ethical standards of the responsible committee on human experimentation (institutional and national) and with the Helsinki Declaration of 1975, as revised in 2008.

### 2.3 Biologic material and ddPCR procedure

Study material consisted of 20 mL of PB collected in EDTA tubes and up to 6 mL of BM, when aspiration was possible. All patients underwent BM trephine biopsy both before and after treatment, which was required to assess the depth of response to treatment by means of May‐Grünwald‐Giemsa staining. Patients in follow‐up with a long‐lasting CR did not receive any marrow sampling, but only PB collection.

**FIGURE 1:**
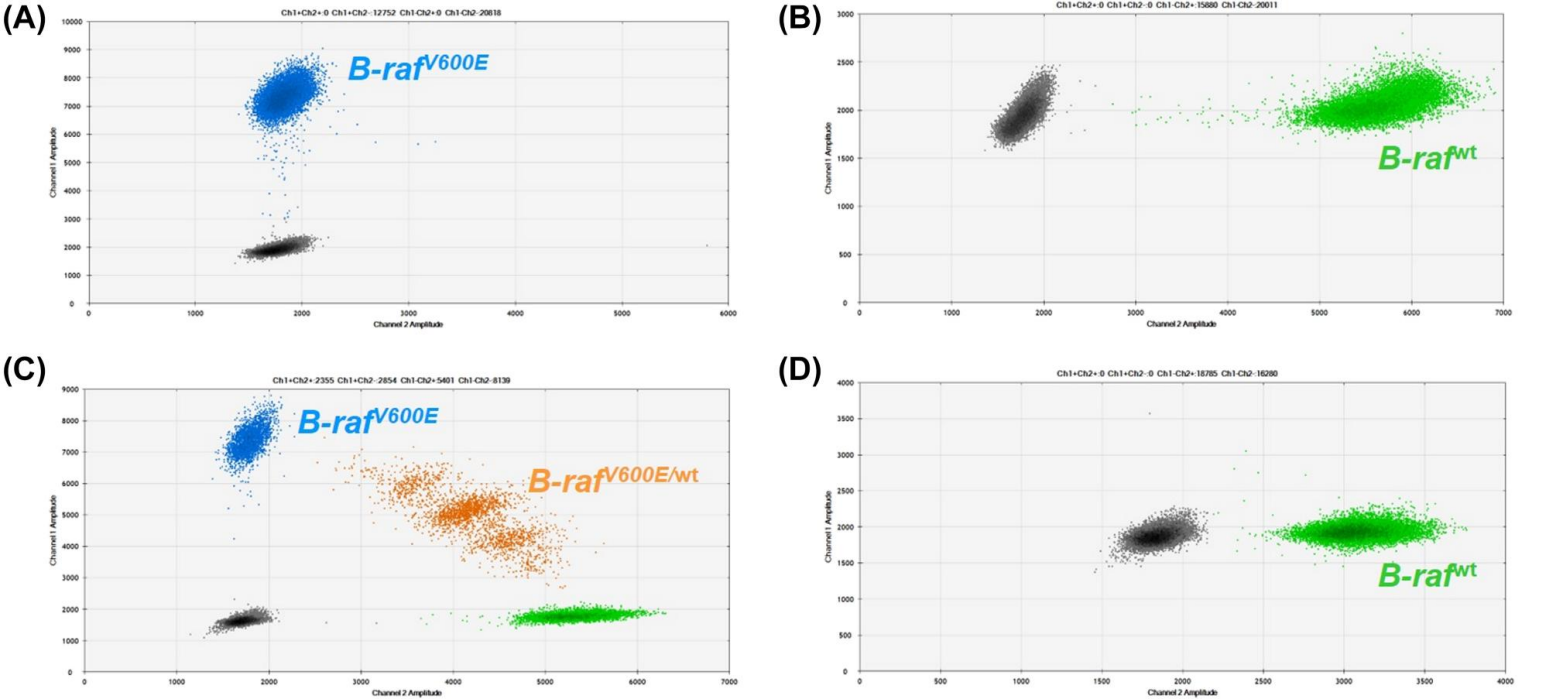
Positive control, *BRAF* ^V600E^‐positive A375 melanoma cell‐line (panel A). Negative control, *BRAF* wild‐type HL60 acute myeloid leukemia cell‐line (panel B). *BRAF* ^V600E^‐positive HCL patient at disease onset, peripheral blood (panel C). An HCL patient with long lasting complete response, peripheral blood (panel D)

PB and BM mononuclear cells have been separated according to a density gradient, then suspended in guanidine isothiocyanate (GITC) and refrigerated at −20°C until analysis. DNA extraction was performed automatically with the Maxwell^®^ 16 LEV Blood Kit (Promega). GITC‐suspended cells have been treated with proteinase K at 56°C for 20 min. DNA separation was performed with Maxwell^®^ 16 System (Promega) and DNA quantitation was done with Nanodrop^TM^. DNA samples have been diluted to a final concentration of 30 ng/µL, then partitioned into droplets by the QX200^TM^ Droplet Generator. Each droplet contained 3 µL of DNA, fluorescent primers and probes for both wild‐type and mutated allele (ddPCR Mutation Detection Assays, Bio‐Rad), nucleotides and polymerase (Bio‐Rad). Once droplets have been generated and transferred into wells, a polymerase chain amplification reaction took place within each droplet, according to the presence of wild‐type or mutated allele, or both. At the end of the reaction, droplets have been read by the QX200 Droplet Reader (Bio‐Rad) and subdivided according to their content: empty droplets, droplets containing a wild‐type allele or a mutated allele, droplets containing both. Positive and negative controls were represented by the *BRAF* ^V600E^‐positive A375 melanoma cell‐line and the *BRAF* wild‐type HL60 acute myeloid leukemia cell‐line, respectively (Figure 1).

Statistical analysis and application of the Poisson's algorithm was performed by QuantaSoft software (Bio‐Rad), and results have been reported as fractional abundance (FA) of the mutated allele.

## 3 RESULTS

### 3.1 Patients and samples

All consecutive patients treated at our institution between October 2015 and March 2019 and receiving frontline treatment with cladribine or salvage treatment with either purine analogs or biologic agents have been considered eligible for laboratory testing. 14 patients have been evaluated at disease onset: 12 of them had their PB tested, and in 4 cases the BM was evaluated. Seven patients have been studied at the time of relapse: measurements were performed on PB in all of them and on BM in six of them. Seven patients have been evaluated at response: importantly, we have selected only patients achieving a CR, as defined by the Consensus Resolution criteria (see Supporting Information).^14^ All the patients evaluated at response have been also tested immediately before treatment, so that paired analyses could be available. Table 1 summarizes the typology of patients tested and the site of sampling. In addition, we have also tested 14 patients who have previously achieved a complete remission after only one line of treatment with cladribine, provided their duration of response was longer than 5 years. In total, 36 patients have been tested: unsurprisingly, 97% of them were males. The overall number of measurements was 56, given that many patients were evaluated more than once (with assays on both PB and BM, and at disease onset and response).

**TABLE 1:** Timing of measurement and sample characteristics

Number of patients	36
Male patients, %	97
Overall number of measurements, n	56
Patients in CR for more than 5 years, *n*	14^a^
Molecularly negative in peripheral blood	10
Patients evaluated at diagnosis, *n*	14
Peripheral Blood/bone marrow	12/4
Patients evaluated at disease relapse, *n*	7
Peripheral Blood/bone marrow	7/6
Patients evaluated at CR (*) after treatment, *n*	7^b^
Peripheral Blood/bone marrow	6/6
Molecularly negative in peripheral blood	4^c^
Patients with pre + post‐therapy evaluation, *n*	7
Patients Evaluated at first diagnosis	5^d^
Patients Evaluated at disease relapse	2^e^

Abbreviation: CR, complete response.

^a^ According to the Consensus Resolution criteria.

^b^ Patients with symptomatic disease (first diagnosis or relapse), always evaluated also before treatment.

^c^ These are patients in complete molecular response after first‐line treatment with cladribine.

^d^ Treated with upfront subcutaneous cladribine.

^e^ Treated at disease relapse with repeated cladribine (1 patient) and moxetumomab pasudotox (1 patient).

### 3.2 FA is descriptive of tumor burden at each disease phase

Mean FA values (±standard deviation) in PB for patients at diagnosis, relapse and with CR were 12.26% (±12.70), 16.52% (±17.11) and 0.02% (±0.03), respectively, and ranged between 0.47% and 37.90%, 1.07% and 51.30%, 0.00 and 0.08% for each aforementioned disease phase (Table 2). Likewise, mean FA in BM, when available, was 23.51% (±13.58), 13.96% (±5.98) and 0.26% (±0.30), for patients at disease onset, relapse and CR, respectively, ranging between 8.92% and 41.60%, 8.05% and 21.10%, 0.03% and 0.79% for each disease phase. Mean FA in PB for the 14 patients with long lasting complete response was 0.05% (±0.10), ranging between 0.00 and 0.31%.

**TABLE 2:** BRAF^V600E^ allele burden expressed as mean fractional abundance at each disease phase, according to sampling site

Disease onset, peripheral blood (*n* = 12)	12.26%
Disease onset, bone marrow (*n* = 4)	23.51%
Relapse, peripheral blood (*n* = 7)	16.52%
Relapse, bone marrow (*n* = 6)	13.96%
Complete response, peripheral blood (*n* = 6)^a^	0.02%^b^
Complete response, bone marrow (*n* = 6)^a^	0.26%
Long responders (*n* = 14)^c^	0.05%

^a^ Complete response is defined according to Consensus Resolution criteria.

^b^ Four patients with complete molecular response.

^c^ Patients in continuous complete response (≥5 years) according to Consensus Resolution criteria.

**FIGURE 2:**
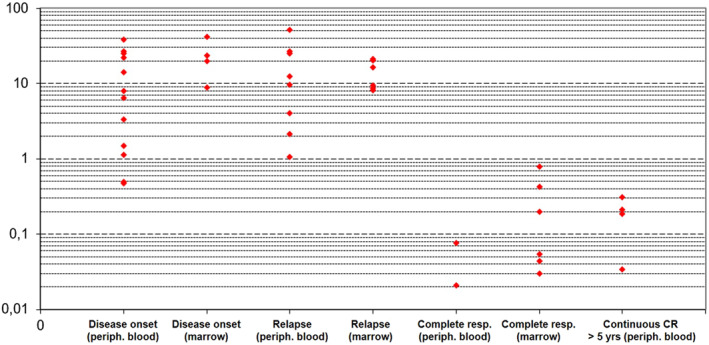
*BRAF* ^V600E^ allele burden expressed as fractional abundance at each disease phase, according to sampling site. Vertical axis is in logarithmic scale. Note that the logarithmic scale does not show patients whose fractional abundance is zero

Point values for each of the performed assay are represented in Figure 2 (with vertical axis logarithmic scale) and in Figure S1 (linear axis). Measured FA of *BRAF* ^V600E^ varies considerably when patients with active disease and patients in CR are considered. In particular, FA values are almost overlapping between patients at onset and at relapse, while patients in CR according to the Consensus Resolution criteria may still display a positive *BRAF* ^V600E^ burden, but generally below 1%. Some patients, instead, are indeed molecularly negative.

Among the seven patients tested both before and after treatment and achieving a CR, the logarithmic reduction of *BRAF* ^V600E^ FA was 2.48 and 1.47 in PB and BM, respectively.

### 3.3 Patients in CR can achieve BRAF^V600E^ negativity in PB

Four patients out of 6 evaluated at response after first‐line treatment with subcutaneous cladribine were molecularly negative for *BRAF* ^V600E^ in PB, while none of the 6 BM samples obtained at response was negative (see paragraph above).

Among the 14 patients with a continuous CR after one course of cladribine who maintained a treatment‐free interval of at least 5 years, 10 (71.4%) were *BRAF* ^V600E^ negative in PB. The four patients with a positive assay displayed a FA of 0.03%, 0.19%, 0.21% and 0.31%. Only one long‐responding patient with a negative assay showed disease progression and required further treatment within 2 years from testing and more than 8 years after frontline cladribine. The patient whose FA was 0.31% displayed symptomatic progression 2 years after testing, 14 years after frontline treatment. At the time of the clinical and histological documentation of relapse, the FA on PB has risen to 1.45%.

### 3.4 Patients with no response to therapy remain BRAF^V600E^‐positive

Besides assays performed on patients in CR after treatment, three patients lacking response have also been tested as a positive control. All of them had a *BRAF* ^V600E^ proven positivity at the time of diagnosis or relapse (thus are included in the data presented above). One of them was tested on both PB and BM, displaying a FA of 9.35% and 21.07%, respectively, after failing cladribine and rituximab. A second patient maintained a FA of 2.46% on BM after the failure of repeated lines of cladribine, and ultimately died of HCL. The third patient showed progressive disease after an initial treatment with subcutaneous cladribine, with a FA on BM of 10.33%: concomitantly, he developed a *BRAF* ^V600E^‐positive monomorphic epitheliotropic intestinal T‐cell lymphoma, which caused his death a few months later.^19^

## 4 DISCUSSION

The Consensus Resolution criteria, along with recently updated guidelines for the diagnosis and management of HCL patients, rely on the resolution of all peripheral cytopenias and organomegaly, as well as on the disappearance of marrow lymphoid infiltration with hematoxylin‐eosin staining, to establish the status of CR.^14^, ^15^ Immunohistochemistry may be a strategy to assess the persistence of a minimal marrow infiltrate: however, neither thresholds for positivity, nor the most appropriate immunohistochemical markers have been validated or widely acknowledged.^13^, ^17^, ^20^, ^21^ Likewise, flow cytometry appears a useful tool, but no consensus is established on which antibody panel needs to be used.^12^, ^22^, ^23^ The application of molecular techniques is still matter of research.

The integration of the standardly defined CR category with MRD data assessment–regardless the method used–may help stratify patients in CR better: in other words, it can be hypothesized that patients in CR with persistent MRD may have a different long‐term prognosis than CR patients with negative MRD findings.^23^, ^24^ Importantly, it is well known that some patients achieve and maintain a long‐term response after only one treatment line, although the factors predicting such a good prognosis are still unknown.^18^ It is much likely that long‐term responders have achieved a much more profound response than what obtained by those suffering of early relapse, albeit categorized in any case as complete responders. In other words, the achievement of an MRD‐negative status may translate into better prognostic rates and much longer treatment‐free intervals.

Our preliminary results suggest that ddPCR permits to assess the active tumor burden in HCL at different stages of disease and support the hypothesis that some patients in CR qualify for a complete molecular response. The *BRAF* ^V600E^ quantified as FA is a good descriptor of disease burden at each phase of HCL natural history and discriminates between patients with active disease (i.e. those at onset and relapse, as well as those with less than CR after therapy), patients in CR with MRD and patients in CR free of the disease.

Beyond its utility in disease diagnosis, as proved by previous literature reports,^9^, ^10^, ^11^ such an assay can be applied for: (i) response categorization, as it may integrate the traditional CR status with the addition of the molecular status of the patient, either *BRAF* ^V600E^ positive or negative; (ii) relapse assessment, in case of peripheral cytopenias not unequivocally attributable to an overt HCL recurrence; (iii) non‐invasive follow‐up in patients after active treatment, with possible influence on the prediction of a symptomatic disease relapse; (iv) evaluation of long‐term responding patients, who may be considered cured in case of a negative assay, provided their follow‐up is sufficiently long.

We found the FA value of 1% represents the threshold to discriminate between active disease (with need of treatment) and CR. In other terms, basing on our data it emerges that all patients with active disease display an FA > 1%; conversely, patients in CR display an FA < 1%, with some of them being molecularly negative. None of the patients with active disease was *BRAF* ^V600E^ negative in our case series.

Although preliminary, these results offer a wider interpretation of the response status obtained after active treatment. The values of FA obtained can be integrated coherently in patients' clinical context, along with their PB counts and BM findings. Besides that, this study has some limitations. First, data are not conclusive on the comparability of PB and BM. This is mainly due to the small number of observations and to the fact that BM is not always easily aspirated in HCL because of reticulin fibrosis. Our data show a persistent minimal positivity of marrow *BRAF* ^V600E^ burden in CR patients, although markedly below the 1% FA threshold, suggestive of an incomplete clearance of BM infiltration. Secondly, we are unable to provide a clear interpretation of FA results falling in close proximity of 1%. These may refer to patients much likely affected by active–although possibly not symptomatic–disease and need to be contextualized within the global clinical history and integrated with more data (e.g. closely repeated PB counts). Third, we lack the initial *BRAF* mutational status of all long‐term responding patients, although they are supposed to be positive in the vast majority due to the extremely high incidence of the mutation in the HCL population at diagnosis.^1^, ^3^ Lastly, paired data (i.e. *BRAF* mutational status before and after treatment) are available in seven patients only, corresponding to those, who received treatment during the study time window.

These preliminary results suggest that ddPCR permits to assess the active tumor burden in at different disease phases and support the hypothesis that some patients in CR can reach a complete molecular response. Further studies are required in order to follow patients prospectively by sampling serially PB and BM at each stage of the disease and to provide validation of this technique.

## CONFLICT OF INTERESTS

The authors have stated that they have no conflict of interest.

## AUTHOR CONTRIBUTIONS

Alessandro Broccoli, Carolina Terragna, and Pier Luigi Zinzani contributed to the design of the study and to work concept. Lisa Argnani performed statistical analyses. Carolina Terragna, Marina Martello, Silvia Armuzzi, Claudio Agostinelli and Elena Sabattini performed lab tests and collect lab data. Alessandro Broccoli, Vittorio Stefoni, Lisa Argnani, Alice Morigi, MC, Beatrice Casadei, Laura Nanni, Cinzia Pellegrini, Carolina Terragna, Marina Martello, Silvia Armuzzi, Claudio Agostinelli, Elena Sabattini and Pier Luigi Zinzani collected clinical data and contributed to data interpretation. All authors read and approved the final manuscript.

### TRANSPARENT PEER REVIEW

The peer review history for this article is available at https://publons.com/publon/10.1002/hon.2932.

## Supporting information

Supporting Information S1

Figure S1

## Data Availability

The data that support the findings of this study are available from the corresponding author upon reasonable request.
