# Supplementary material for: Droplet digital polymerase chain reaction for the assessment of disease burden in hairy cell leukemia
Source: Hematol Oncol. 2021 Oct 15;40(1):58–63. doi: 10.1002/hon.2932 (PMC9291464; doi:10.1002/hon.2932)
Supplement: Supplementary file 1 — Supporting Information S1 [file HON-40-58-s002.docx]

**SUPPLEMENTARY MATERIAL**

**Droplet digital polymerase chain reaction for the assessment of disease burden in hairy cell leukemia.**

Alessandro Broccoli^1,2^, Carolina Terragna^1^, Laura Nanni^1,2^, Marina Martello^1,2^, Silvia Armuzzi^1,2^, Claudio Agostinelli^1,2^, Alice Morigi^1,2^, Beatrice Casadei^1,2^, Cinzia Pellegrini^1^, Vittorio Stefoni^1,2^, Elena Sabattini^3^, Lisa Argnani^2^, Pier Luigi Zinzani^1,2^

^1^IRCCS Azienda Ospedaliero-Universitaria di Bologna, Istituto di Ematologia “Seràgnoli”, Bologna, Italy.

^2^Dipartimento di Medicina Specialistica, Diagnostica e Sperimentale, Università di Bologna, Bologna, Italy

^3^Hematopathology Unit, IRCCS Azienda Ospedaliero-Universitaria di Bologna, Bologna, Italy.

**SUPPLEMENTARY METHODS**

The Consensus Resolution Criteria published in 1987, define complete response as the resolution of all peripheral blood cytopenias (with hemoglobin being at least 12 g/dL, platelet counts higher than 100,000/mmc and neutrophil counts higher than 1,500/mmc), along with the disappearance of any circulating hairy cells and the absence of organomegaly. No residual hairy-cell infiltrate needs to be detected upon bone marrow histology, when specimens are stained with hematoxylin-eosin. Partial response requires the resolution of all peripheral blood cytopenias, with the persistence of at least 5% of circulating hairy cells or the reduction of at least 50% of the bone marrow leukemic infiltrate or of any previously detected organomegaly. Minor (or minimal) response indicates a peripheral blood hematological improvement in at least one among hemoglobin concentration, platelet counts, neutrophil counts, without the clearance of circulating hairy cells (yet being less than 50% of the initial circulating burden). Non-responder patients are those who fail to achieve at least a minor response.

**SUPPLEMENTARY FIGURE LEGEND**

**Figure S1. *BRAF*^V600E^ allele burden expressed as fractional abundance at each disease phase, according to sampling site. Vertical axis is in linear scale.**
