# Supplementary figures and images for: Droplet digital polymerase chain reaction for the assessment of disease burden in hairy cell leukemia
Source: Hematol Oncol. 2021 Oct 15;40(1):58–63. doi: 10.1002/hon.2932 (PMC9291464; doi:10.1002/hon.2932)

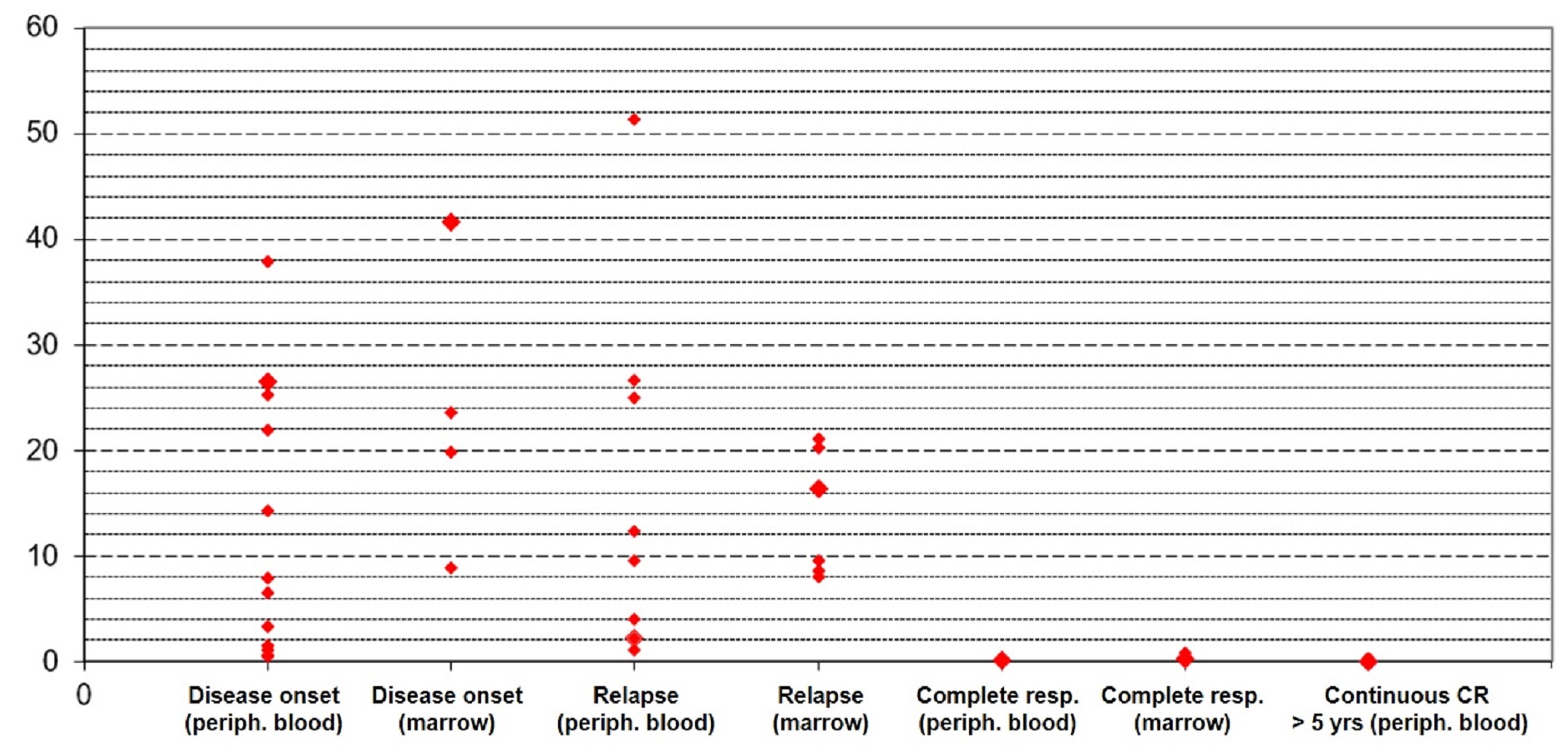

Supplement: Supplementary file 2 — Figure S1 [file HON-40-58-s001.jpg]
